# Uniportal Full Endoscopic Posterolateral Transforaminal Lumbar Interbody Fusion with Endoscopic Disc Drilling Preparation Technique for Symptomatic Foraminal Stenosis Secondary to Severe Collapsed Disc Space: A Clinical and Computer Tomographic Study with Technical Note

**DOI:** 10.3390/brainsci10060373

**Published:** 2020-06-15

**Authors:** Pang Hung Wu, Hyeun Sung Kim, Yeon Jin Lee, Dae Hwan Kim, Jun Hyung Lee, Jun Bok Jeon, Harshavardhan Dilip Raorane, Il-Tae Jang

**Affiliations:** 1 Spine Surgery, Nanoori Gangnam Hospital, Seoul 06048, Korea; pang_hung_wu@nuhs.edu.sg (P.H.W.); nigaheboa@gnnanoori.co.kr (Y.J.L.); dhkim225@daum.net (D.H.K.); drbrainlee@gmail.com (J.H.L.); godisma@gnnanoori.co.kr (J.B.J.); dr.harsh35@gmail.com (H.D.R.); nanooriresearch@gmail.com (I.-T.J.); 2 National University Health System, JurongHealth Campus, Orthopaedic Surgery, Singapore 609606, Singapore

**Keywords:** endoscopic spine surgery, transforaminal lumbar interbody fusion, degenerative spine disease, endoscopic lumbar interbody fusion, spinal fusion

## Abstract

**Background:** Severe collapsed disc secondary to degenerative spinal conditions leads to significant foraminal stenosis. We hypothesized that uniportal posterolateral transforaminal lumbar interbody fusion with endoscopic disc drilling technique could be safely applied to the collapsed disc space to improve patients’ pain score, restore disc height, and correct the segmental angular parameters. **Methods:** We included patients who met the indication criteria for lumbar fusion and underwent uniportal full endoscopic posterolateral transforaminal lumbar interbody fusion with pre-operative Computer Tomography mid disc height of less than or equal to 5 mm and MRI of Grade 3 Foraminal Stenosis. Visual analogue scale and computer tomography pre-operative and post-operative sagittal disc height in the anterior, middle and posterior part of the disc; sagittal focal segmental angle; mid coronal disc height and coronal wedge angles were evaluated. **Results:** 30 levels of Endo-TLIF were included, with a mean follow up of 12 months. The mean improvement in decreasing pain score was 2.5 ± 1.1, 3.2 ± 0.9 and 4.3 ± 1.0 at 1 week post operation, 3 months post operation and at final follow up, respectively, *p* < 0.05. There was significant increase in mid sagittal computer tomographic anterior, middle and posterior disc height of 6.99 ± 2.30, 6.28 ± 1.44, 5.12 ± 1.79 mm respectively, *p* < 0.05. CT mid coronal disc height showed an increase of 7.13 ± 1.90 mm, *p* < 0.05. There was a significant improvement in the CT coronal wedge angle of 2.35 ± 4.73 and the CT segmental focal sagittal angle of 1.98 ± 4.69, *p* < 0.05. **Conclusion:** Application of Uniportal Endoscopic Posterolateral Lumbar Interbody Fusion in patients with severe foraminal stenosis secondary to severe collapsed disc space significantly relieved patients’ pain and restored disc height without early subsidence or exiting nerve root dysesthesia in our cohort of patients.

## 1. Introduction

**Figure 1:**
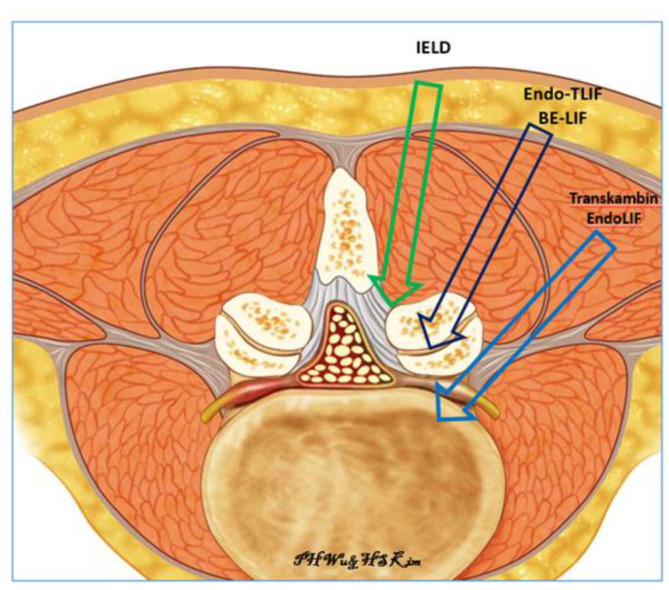
Three routes of Endoscopy. IELD is interlaminar endoscopic lumbar discectomy, Endo-TLIF is uniportal full endoscopic posterolateral transforaminal lumbar interbody fusion, BE-LIF is biportal endoscopic lumbar interbody fusion, Transkambin EndoLIF is uniporal transforaminal transkambin approach endoscopic lumbar interbody fusion.

The aging of the population has led to an increased incidence of symptomatic degenerative spinal conditions such as degenerative spinal stenosis, spondylolisthesis and degenerative disc disease [1]. In carefully selected patients, spinal fusion has shown good results as a method of management in the treatment of degenerative spinal conditions [2,3,4]. Advancements in endoscopic spine surgery techniques have led to more minimally invasive options for lumbar spine surgery [5]. Techniques using full endoscopic interlaminar ipsilateral and contralateral approaches evolved from discectomy to decompression in order to address various degenerative spinal pathologies in the central, lateral recess and extraforaminal regions [6,7,8,9]. One of the more recent challenges in lumbar endoscopy is endoscopic assisted fusion. There are two common posterior routes for endoscopic interbody fusion described in the literature. The first route is the transforaminal endoscopic approach using the safe corridor of Kambin’s Triangle, ventral to the facet joint; this route is used in the Uniportal Endoscopic Transkambin Facet Sparing Approach for transforaminal lumbar interbody fusion. Several authors have had good clinical results with this technique using an expandable cage [10,11,12]. The second route is the posterolateral approach for performing transforaminal lumbar interbody fusion. This route explores the pathway described by Harms et al. for open transforaminal lumbar interbody fusion [13]. Currently, this approach is mainly used by surgeons who practice biportal endoscopic assisted posterolateral lumbar interbody fusion [14,15]. There is paucity of literature describing the uniportal endoscopic posterolateral approach for transforaminal interbody fusion (Endo-TLIF). Kim and Wu et al. first described the use of the uniportal full endoscopic approach to perform posterolateral transforaminal lumbar interbody fusion in a case report in which they completely resected the ipsilateral facet joint and introduced a large interbody cage to reduce the translational deformity in a grade 2 spondylolisthesis patient [16]. In this study, we expanded the indication of this technique, uniportal full endoscopic posterolateral transforaminal lumbar interbody fusion (Endo-TLIF), described by Kim and Wu et al., in order to treat patients with symptomatic foraminal stenosis secondary to severe collapsed disc height. The hypothesis was that this technique could significantly increase the intervertebral disc height and relieve the leg pain caused by foraminal stenosis. In this technical note, we describe our early experience with this technique and evaluate the computer tomographic results and clinical outcomes for a selected cohort of patients (Figure 1).

## 2. Materials and Methods

### 2.1. Indication, Inclusion and Exclusion Criteria

This retrospective study was approved by institutional review board of Nanoori Hospital, Seoul, Republic of Korea, NR IRB number 2020-016. Informed consent was obtained from all patients participating in the study.

**Figure 2:**
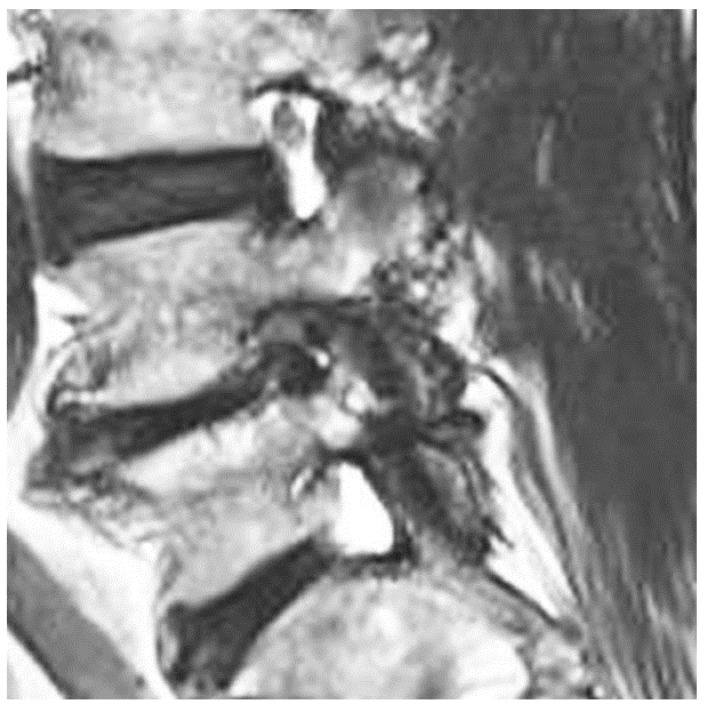
Mid right foraminal MRI of lumbar spine. It showed severe grade 3 Lee et al. foraminal stenosis with morphological change of right L4 exiting nerve root in right L4/5 foramen.

Nanoori Hospital maintained a prospectively collected database of all patients who underwent Endo-TLIF. PHW, YJL and DHK retrospectively reviewed all radiological and clinical data from the database. From the Endo-TLIF data base, we included symptomatic patients who suffered back pain and lumbar claudication who failed a minimum of 6 weeks of conservative treatment. They had the following clinical diagnoses: (1) grade 2 and below spondylolisthesis; (2) spinal stenosis with instability; (3) end-stage degenerative disc disease with severe foraminal stenosis. They had radiological parameters as follows: (1) severe disc collapse with mid sagittal lumbar spine Computer Tomographic (CT) middle disc height shorter than 5 mm; and (2) Magnetic Resonance Imaging (MRI) of Grade 3 Classification of Foraminal Stenosis by Lee et al. [17] (Figure 2). We excluded patients who had spinal fusion surgery due to trauma, revision surgery, tumor, infection, pseudarthrosis, congenital spinal deformity, sagittal malalignment and coronal deformity with more than a 20-degree coronal curve. 

**Figure 3:**
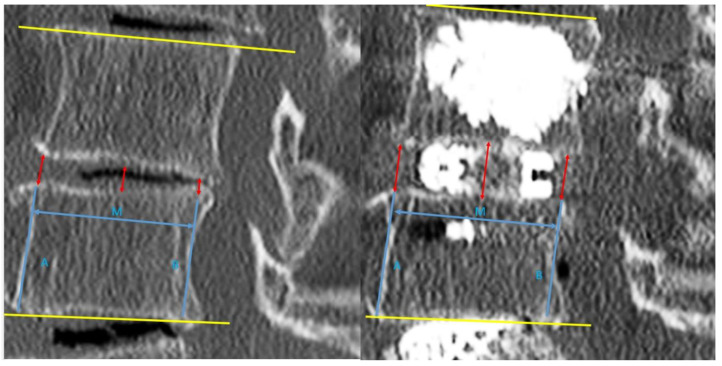
Mid sagittal cut of CT lumbar spine for measurement of parameters with left picture showed pre-operative cut and right picture showed post-operative cut of mid sagittal CT lumbar spine. Anterior vertebral line was drawn for caudal vertebra (line A) and anterior disc height was measured from the line parallel to line A from the caudal end plate to the cephalad end plate. The posterior vertebra line was drawn for caudal vertebra (line B) and posterior disc height is measured from line parallel to line A from caudal end plate to cephalad end plate. Middle disc height is measured at the end plate in the midpoint of lines A and B (M point) to the corresponding cephalad vertebral body end plate.

**Figure 4:**
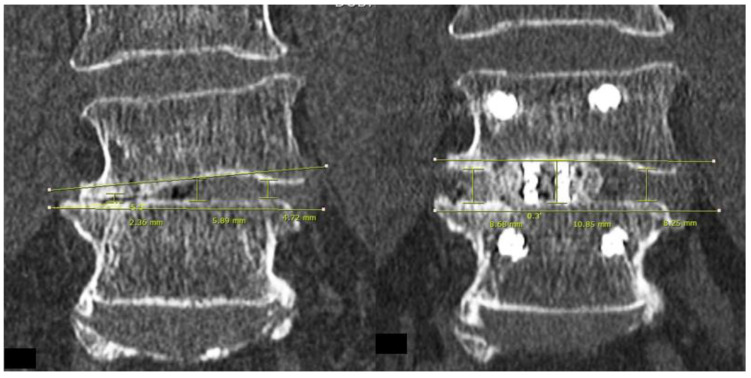
Left picture was pre-operative and right picture was post-operative mid Coronal Cut of CT Lumbar Spine of L4/5, we measured the pre-operative and post-operative mid Coronal Disc height and Coronal Wedge Angle.

For post-operative evaluation in this cohort of patients, we evaluated clinical outcomes of Visual Analogue Scale at pre-operative, 1 week post-operative, 3 months post-operative and final follow up. Early and late perioperative complications were documented. For radiological evaluation, CT lumbar spine evaluation was done on pre-operative and post-operative day one. We evaluated mid sagittal CT lumbar spine to measure anterior, middle and posterior disc height and focal segmental angle (Figure 3). CT evaluation at mid coronal lumbar spine was done to measure mid coronal disc height and coronal wedge angle (Figure 4). 

### 2.2. Surgical Technique

#### 2.2.1. Pre-Operative Preparation

After a detailed history and clinical examination, each patient underwent anteroposterior, lateral, flexion and extension view of the lumbar spine to assess for radiographic parameters of spinal instability. In the pre-operative lateral view standing, flexion and extension lumbar XR, we measured the sagittal parameters, and evaluated for radiological signs of instability in the patient, with flexion extension radiographs showing sagittal plane translation of >4.5 mm and/or relative sagittal plane angulation >22° [18]. A 3-foot roentgenogram was done to evaluate sagittal balance and coronal balance. For the purposes of the study, we excluded cases with coronal deformity of more than 20 degrees and those cases who had sagittal imbalance. Magnetic Resonance Imaging (MRI) was performed to assess for levels and degree of stenosis, in this study, we only included grade 3 severe foraminal stenosis with nerve root morphologic change secondary to compression, in accordance with Lee et al. [17]. Computer Tomography (CT) scan was performed to evaluate the disc height parameters, the focal segmental angle and coronal segmental wedge angle and also evaluate the morphology of pedicles [19]. 3D reconstruction CT scan was helpful in giving the surgeon a visual impression of the amount of bone resection needed. The operative side was decided based on the side of the main symptoms. Nerve root block and conservative treatments were discussed as other options to every one of the patients, the patients opted to undergo Endo-TLIF in this cohort. 

#### 2.2.2. Anesthesia and Skin Incision

**Figure 5:**
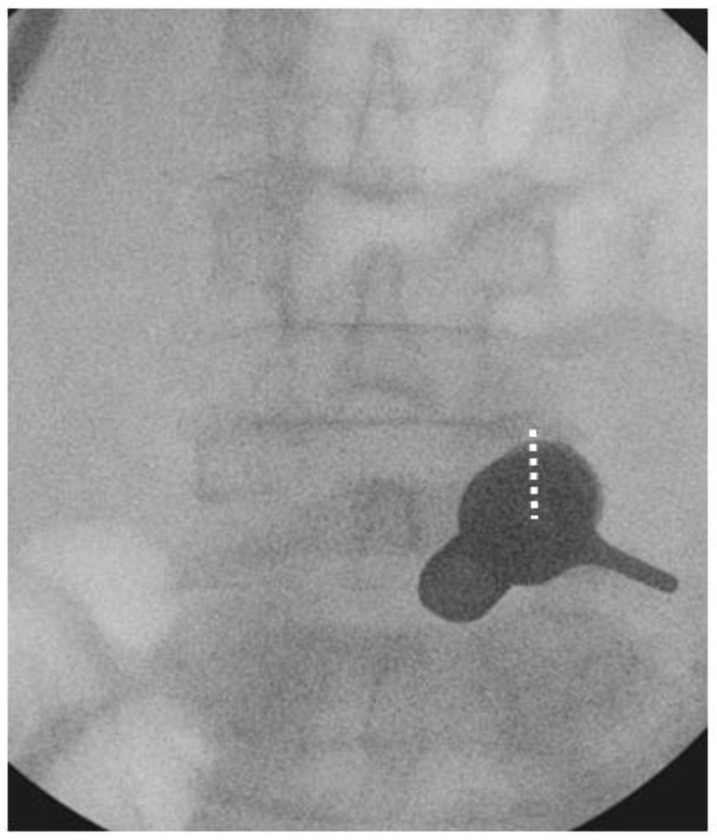
Docking position of right L4/5 Endo-TLIF. The incision was made over the right L4 pedicle (dotted white line) and the working cannula was docked on the pars interarticularlis of right L4/5.

We performed the procedure under general anesthesia. The patient was positioned in a prone position on a Wilson Frame over a radiolucent operating table with the spine in slight flexion. The endoscopic procedure was performed with an irrigation fluid pressure of 25–40 mmHg using an irrigation pump. We stood on the symptomatic side and used the upper pedicle screw skin incision for the uniportal endoscope insertion and docking for each corresponding level of posterolateral approach interbody fusion. For example, we used the skin incision over the right L4 pedicle on the AP intraoperative fluoroscopic image to perform right L4/5 Endo-TLIF (Figure 5) We incised a 1.6 cm skin incision to facilitate working cannula placement and made an extended fascia incision of 3 cm to allow mobility of the working channel and subsequent rod placement after completion of interbody cage insertion. We performed the insertion of the cage(s) prior to pedicle screw insertion.

#### 2.2.3. Insertion of Endoscope

Guidewire followed by serial dilators were placed through the skin incision and docked on the isthmus, which was followed by insertion of a 13.7 mm diameter beveled-tip working cannula. We performed an intraoperative anteroposterior and lateral view at this point of time to confirm the correct level of decompression. An endoscope of 15° viewing angle, outer diameter 10 mm, working channel diameter of 6 mm and working length 125 mm was inserted for the surgical procedure.

#### 2.2.4. Surgical Procedure

**Figure 6:**
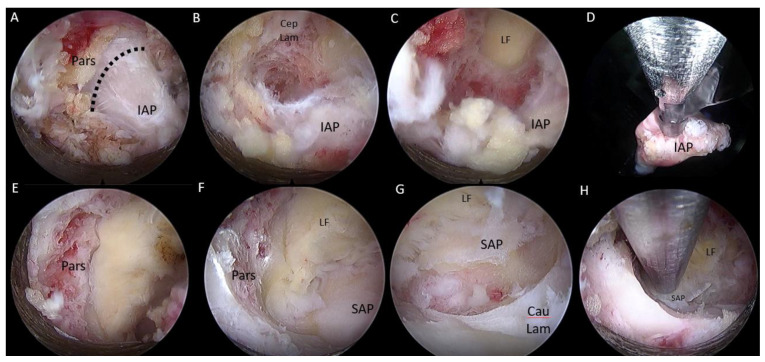
Endo TLIF facet resection was done on left L5/S1. (**A**) Exposure of inferior articular process(IAP) and pars interarticularis(Pars). (**B**) Endoscopic drilling was done at the junction between cephalad lamina (Cep Lam) and IAP. (**C**) Endoscopic drilling exposing the deep layer of ligamentum flavum and last thin cortical layer of IAP. (**D**) IAP was resected and harvested as bone graft. (**E**) Exposure of pars interarticularis which was trimmed with endoscopic drill. (**F**) Exposure of superior articular facet (SAP). (**G**) Endoscopic drilling was done at the base of SAP along caudal lamina (Cau Lam). (**H**) SAP was resected and harvested for bone graft.

**Figure 7:**
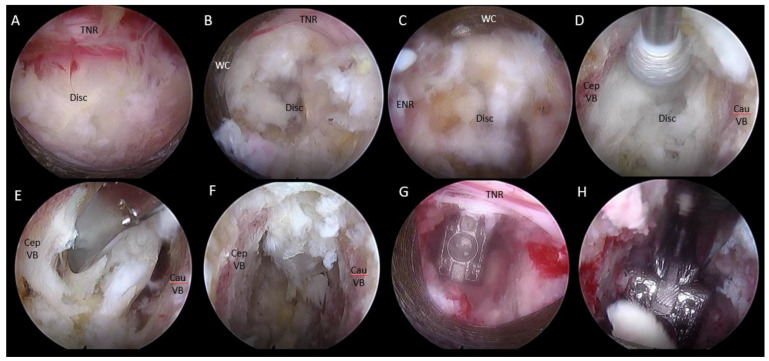
Endo-TLIF disc and end plate preparation of left L5/S1. (**A**) Exposure of L5/S1 disc and traversing nerve root (TNR) after ligamentum flavum removed. (**B**) Working cannula (WC) advanced and rotated to protect the exiting nerve root docking on the disc while making sure traversing nerve root was not caught under the working cannula. (**C**) Working cannula rotated clockwise to protect the traversing nerve root and gently retracting it medially to expose the disc space. (**D**) Endoscopic disc drilling was performed to remove syndesmophytes, disc adhesions and open up intervertebral disc space. (**E**) Disc and cartilage was denuded off the end plate by using mixture of drill, endoscopic probe and forceps. Bone curettes were not used to prevent end plate violation. (**F**) Endplate preparation showed punctate bleeding from endplate. (**G**) Intervertebral cage was inserted and checked that it was in optimal position. (**H**) Cage could be adjusted with punch under endoscopic visualization.

**Figure 8:**
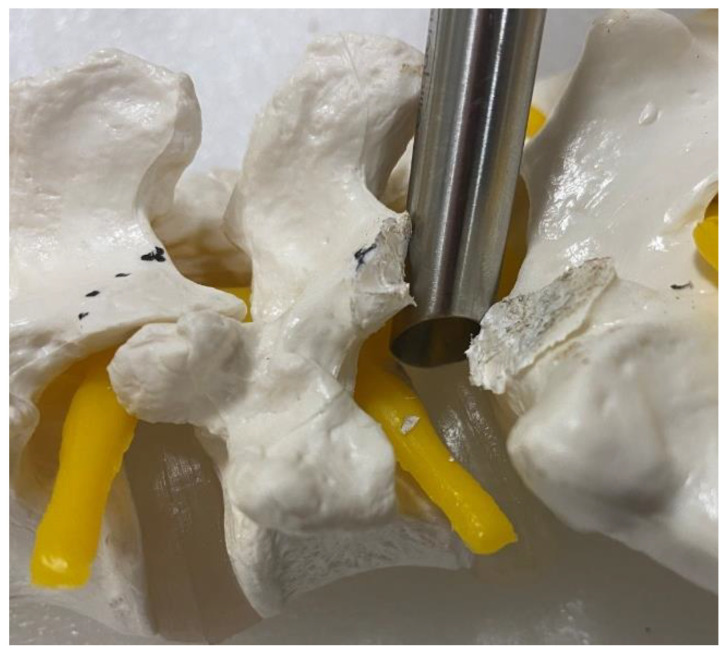
A saw bone model showing working cannula positioned in order to protect the neural elements and open up the posterior border of intervertebral disc space.

**Figure 9:**
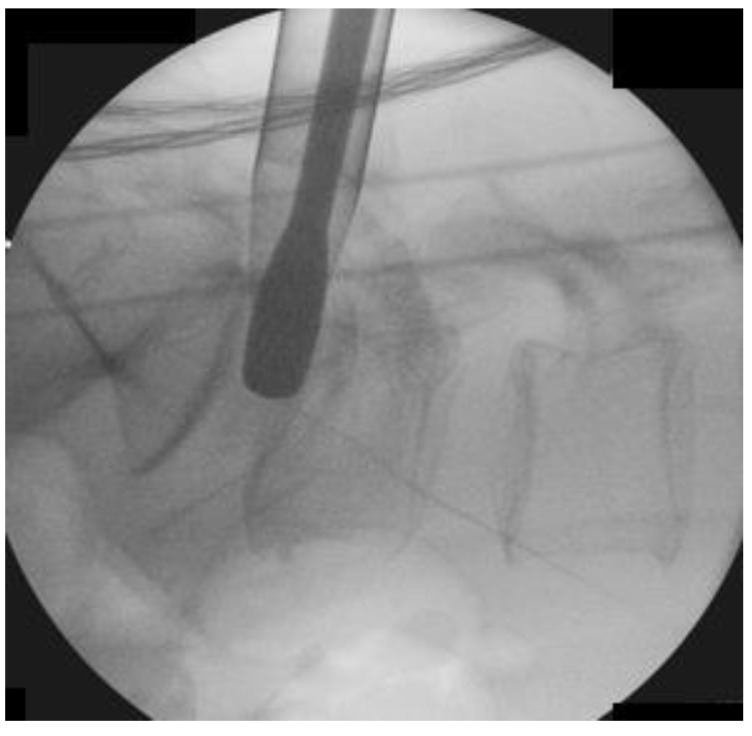
Working cannula was inserted to the posterior third portion of the intervertebral disc space and the disc space was kept apart, allowing an appropriate sized trial to be inserted.

After insertion of endoscope, a clear endoscopic view was obtained by performing hemostasis with radiofrequency ablator. Soft tissue was dissected off the isthmus of the cephalad lamina and the facet joint (Figure 6A) inferior articular process was drilled in a curvilinear direction starting from the spinolaminar junction to superolateral region of inferior articular process (Figure 6A–C). A complete inferior articular facetectomy was performed and harvested as autograft (Figure 6D). Pars interarticularis was trimmed with an endoscopic drill to provide more working space for interbody fusion (Figure 6E). A superior articular process was exposed after the inferior articular facet was removed (Figure 6F). We proceeded to drill the base of the superior articular process from the medial to the lateral direction and remove the superior articular process as an autograft (Figure 6G,H). We removed the underlying ligamentum flavum overlying the disc and neural elements with endoscopic Kerisson rongeur and forceps. There would be epidural vessel bleeding upon removal of ligamentum flavum. We increased the irrigation pressure to 80 mmHg and obtained hemostasis with radiofrequency ablator application on the epidural vessels on the disc space. The radiofrequency ablator would also release adhesion around the degenerative disc and neural structures and exposed the intervertebral disc space. The exiting nerve root could be lying directly over the collapsed disc space. We carefully advanced and rotated the working cannula clockwise with the open bevel pointing away from the exiting and traversing nerve root in order to retract the neural structures away gently, and the disc was isolated within the working cannula (Figure 7A–C). A radiofrequency ablator was used to perform annulotomy on the exposed disc. It was followed by forceps, plasma coagulators, blunt probe and drill in combination to open up the annulus. In a severe collapsed disc space, there would usually be large syndesmophytes or a calcified disc obstructing entry of the disc space. We used a 3.5 mm endoscopic diamond burr to resect the syndesmophyte or calcified disc to open up the disc space (we termed this part of the procedure endoscopic disc drilling) (Figure 7D). We advanced and rotated the working cannula into the opening of the disc space to keep the disc space open (Figure 8). Through the “tunnel” created by the working cannula, we performed end plate preparation for interbody fusion under endoscopic vision using a mixture of endoscopic drill, forceps, a blunt bent probe and a plasma coagulator (Figure 7E). The authors did not use a curette in the process of end plate preparation in order to prevent endplate violation. The optimal fusion bed preparation would be evidenced by denudation of the end plate cartilage with punctate bleeding of subchondral bone (Figure 7F). Once end plate preparation was complete, the working cannula was further advanced into the intervertebral disc space with the tip of working cannula reaching to the dorsal third of the disc space. While the posterior third of the disc is held open by the large working cannula, the anterior portion of the intervertebral disc space might require the use of serial dilators to dilate the disc space gradually to widen the disc space in order to fit an appropriately sized trial (Figure 9). Once the working cannula was in place, the trial was removed and an admixture of autograft and allograft was placed in the ventral and contralateral disc space under fluoroscopic guidance. Finally, a trial was reinserted in order to compact the bone graft and provide guidance for the appropriate size of cage to be inserted with fluoroscopy. Under fluoroscopic guidance, we inserted the appropriate sized 3D-printed titanium cage packed with autograft into the disc space through the same working cannula. The neural elements were gently retracted to provide enough space for cage insertion. After cage insertion was completed under fluoroscopic guidance, the endoscope was inserted to evaluate the position of the cage and the status of neural decompression (Figure 7G). The cage could be adjusted into optimal position under direct endoscopic vision (Figure 7H). Adequate decompression would demonstrate that neural elements were pulsating under irrigation fluid. A drain was inserted under direct endoscopic vision and anchored with a suture. The drain would be removed on day 1, post operation. 

## 3. Pedicle Screw and Rod Insertion with Appropriate Compression and Distraction

**Figure 10:**
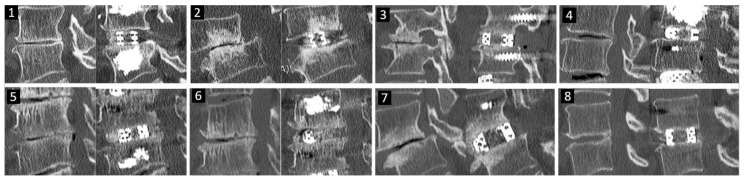
Eight different patients’ pre and post-operative mid sagittal cut CT scan showing the increase in the intervertebral disc height.

**Figure 11:**
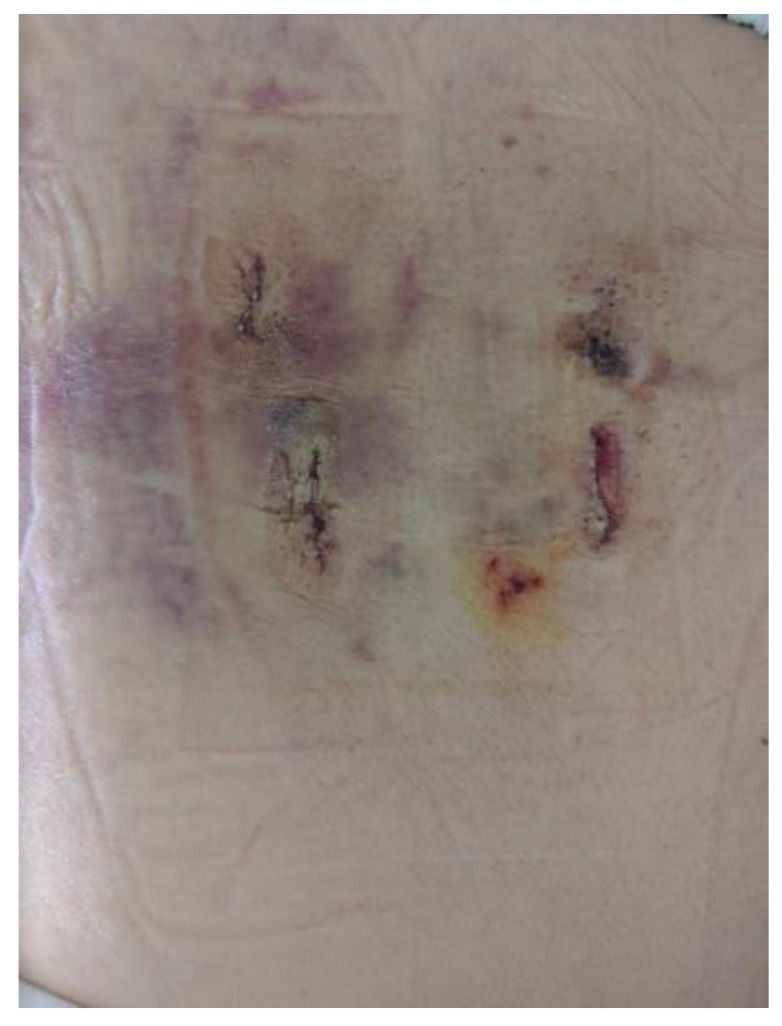
Right L4/5 Endo-TLIF scar on post-operative day seven.

We released the height of the Wilson frame after the cage insertion was completed. Under fluoroscopic guidance, we inserted percutaneous pedicle screws in the standard fashion. If there was a haptic response of low bone density during percutaneous cannulation, cement augmented pedicle screws were inserted. Cement was injected through the percutaneous trochar prior to insertion of self-tapping pedicle screws (Figure 10: Patients 1, 4, 5 and 6). The intervertebral body cage, deployed in a good position, usually restored the disc height. Two bent rods of appropriate length and lordosis were prepared and inserted using the percutaneous rod holder. We performed compression of the pedicle screws and finally tightened the set screws over the rod. The wound was closed in layers (Figure 11).

### 3.1. Post-Operative Care

All Endo-TLIF patients stayed in the general ward after operation. No patient required any high dependency or intensive care unit admission. On post-operative day one, all patients were mobilized and they had CT and standing XR performed as per hospital protocol. None of the patients required opioids or steroids after post-operative day one, so we used NSAIDs and paracetamol for post-operative pain management. Patients were followed up at one week, 3 months, 6 months and 1 year post operation. We reviewed them every six months after one year post operation. 

### 3.2. Statistical Analysis

Data were analyzed with SPSS version 18 statistical analysis software (IBM Corporation, New York, NY, USA). The continuous variables were expressed as mean and standard deviation (SD). The paired *t* test is used for comparison of computer tomography scan (CT scan), pre-operative and post-operative mid-section coronal cut middle disc height, coronal wedge angle, and mid sagittal cut measuring pre- and post-operative anterior, middle and posterior disc height, as well as mid sagittal segmental focal angle. Clinical visual analogue scale (VAS), as measured pre-operatively, 1 day post-operatively, 1 week post-operatively and at final follow up, as reported by the patients, was analyzed with paired *t* test. A value of (*p* < 0.05) was considered significant.

## 4. Results

### 4.1. Baseline Demographics

In the period between October 2018 and October 2019, a total of 60 patients underwent Endo-TLIF. Among these patients, 33 patients had a CT middle disc height greater than 5 mm, and were excluded. Twenty-seven patients with 30 levels of severe collapsed disc space met the inclusion and exclusion criteria. Their mean age was 68 (54–78) years old, with a mean follow up of 12 (6–19) months. There were four levels of degenerative disc diseases with severe foraminal stenosis, two levels with spinal instability without spondylolisthesis on neutral standing XR, and 24 levels of grade 1 spondylolisthesis diagnosis in our 30 levels of Endo-TLIF. There were 9 male and 18 female patients in this cohort. Twenty-four patients had 1 level, and three patients had two levels of Endo-TLIF. Of the three patients with two levels of Endo-TLIF, one patient had L2 to L4, and two patients had L3 to L5 Endo-TLIF. One L2/3, three L3/4, nineteen L4/5 and two L5/S1 single level Endo-TLIF were performed. All the patients underwent general anesthesia for the surgery. 

### 4.2. Clinical and Radiological Outcomes

In terms of clinical evaluation, we had one incidental durotomy during the procedure in 30 levels (3.3%), which required dural patch blocking repair [20]. No revision surgery was required for this patient, who had incidental durotomy without neurological sequelae and was allowed to mobilize post-operatively day two. None of the patients had neurological complications after surgery. No revision surgery was performed. Visual Analog Scale scores measured at pre-operative, 1 week post-operative, 3 months post-operative and final follow up had means and ranges of 6.23 (5–7), 3 (2–5), 3.03 (1–4), and 1.87 (1–3), respectively. There was statistically significant decrease in VAS pain score at 1 week post-operative (2.53 ± 1.14), 3 months post-operative (3.20 ± 0.89) and final follow up (4.37 ± 1.03), respectively, *p* < 0.05.

MRI lumbar scans for all patients with mid sagittal CT lumbar spine mid disc heights of less than 5 mm who had symptomatic foraminal stenosis were pre-operatively evaluated, and coincidentally, all of these patients had grade 3 Lee classification foraminal stenosis (Figure 2) [17].

In terms of radiological results, the position of the implants was satisfactory in all cases. No subsidence of implants was found to have occurred in our cohort at final follow up XR. The means and ranges of pre-operative mid sagittal CT lumbar spine anterior, middle and posterior disc heights were 3.91 (1.71–6.99) mm, 3.8 (0.43–4.98) mm, and 3.84 (0.43–7.03) mm, respectively. The post-operative mid sagittal lumbar spine CT anterior, middle and posterior disc heights were 11.1 (8.18–15.6) mm, 10.2 (7.28–12.4) mm, and 9.12 (5.88–11.5) mm, respectively. The means and ranges of pre-operative and post-operative mid coronal lumbar CT disc height were 3.42 (0.43–4.98) mm, and 10.6 (6.59–14.5) mm, respectively. The pre-operative and post-operative mid coronal lumbar CT wedge angles were 8.02 (0.1°–19.3°) and 5.51 (0°–15.5°), respectively. The pre-operative and post-operative mid sagittal lumbar CT focal segmental angles were 10.9 (−3.4° to 27°) and 12.9 (−1.9° to 31°), respectively.

There was an increase in computer tomographic mid sagittal anterior, middle and posterior disc height of 6.99 (±2.30) mm, 6.28 (±1.43) mm, 5.12 (±1.79) mm, respectively, *p* < 0.05. Similar findings were shown in CT mid coronal disc height, with an increase of 7.13(±1.90) mm, *p* < 0.05. There was a significant improvement in CT coronal wedge angle in post-operative scan compared to the pre-operative scan of 2.35 (±4.73°), and in the mid sagittal CT focal segmental angle of 1.98 (±4.69°), *p* < 0.05. 

Figure 10 shows the mid sagittal CT cuts of eight different patients’ pre- and post-operative CT scans. 

Figure 11 shows the state of the incisions required to perform Endo-TLIF of one level.

Table 1 shows the summary of the radiological and clinical parameter improvements in patients with severe collapsed disc height who underwent Endo-TLIF.

## 5. Discussion 

There has been significant advancement in endoscopic spine surgery techniques for treating degenerative spinal conditions. Lumbar endoscopic surgery treatments ranging from disc herniation, requiring discectomy, to spinal stenosis, requiring bilateral and contralateral endoscopic decompression, have been described with good clinical outcomes [5,8,21,22,23]. There has been a corresponding increase in the clinical application of endoscopic fusion as an option for the treatment of patients with degenerative spinal conditions. The results of endoscopic fusion through uniportal transforaminal transkambin route and biportal-assisted endoscopic fusion have provided positive clinical results [10,14,15,24]. The main advantage of uniportal transforaminal transkambin endoscopic fusion is the ability to perform surgery under local anesthesia with sedation. As uniportal transforaminal transkambin endoscopic fusion operates within a small safe corridor of Kambin’s triangle, which requires a narrow width cage with an expandable cage in order to fit through the small safety corridor, and which is deployed in the intervertebral disc space [10,24,25]. With a narrow width cage used in uniportal transforaminal transkambin endoscopic fusion, there would be higher pressure on the end plates, which may be cause a higher incidence of subsidence, especially if the end plate was violated during end plate preparation. The main advantage of biportal endoscope-assisted interbody fusion is the ability to use large interbody cages to perform interbody fusion with clarity of vision by using an arthroscope during the procedure [14,26]. There is paucity of literature regarding techniques for delivering large interbody cages through uniportal endoscopy. Kim and Wu et al. applied uniportal full endoscopic posterolateral route transforaminal interbody fusion using a similar approach to biportal endoscopic interbody fusion; in their technique, the facets were harvested as bone graft in a patient with grade 2 spondylolisthesis. They used a large interbody cage to reduce the translational deformity with good clinical results [16]. We used this technique to perform interbody fusion in patients with severe collapsed disc space in our study. Using a large interbody cage with a large footprint places less pressure on the vertebral endplate. Endo-TLIF was shown in our series to have less likelihood of subsidence of the interbody cage, which is more frequently encountered in uniportal transforaminal transkambin endoscopic fusion. 

The complete resection of the ipsilateral facet joint through Endo-TLIF created enough space for the large interbody cage used in microscopic tubular transforaminal lumbar interbody fusion. The added advantages of the use of a large interbody cage include (1) good disc height and focal segmental angles correction using this technique; (2) versatility in using various commercially available TLIF cages as compared to the specific requirement of expandable cages used in tranforaminal endoscopic approach via Kambin’s triangle; (3) more bone graft could be packed in both the cage and the disc space to promote bone fusion. 

A large interbody cage is better able to stabilize the anterior column of the lumbar spine, a concept that the authors believe is important as a load-sharing principle [27]. This promotes fusion and prevents implant failure due to reversed bending movement generated if there is insufficient anterior column support. 

In comparison to the transforaminal transkambin facet sparing approach, which typically prepares the end plate under fluoroscopy with intermittent endoscopic inspection, we prepared the end plates under direct endoscopic vision throughout the entire process of end plate preparation in Endo-TLIF. We looked out for the punctate bleeding of end plates, which could be seen without any breach of the cortex of the end plate. No subsidence occurred in our series, compared to the 6% rate of subsidence resulting from transkambin transforaminal interbody fusion [10]. 

With severe collapsed disc space, the natural corridor of Kambin’s triangle in a narrowed lumbar foramen is even smaller due to the proximity of the exiting nerve root to the disc space, which could explain the reason for the relatively higher rate of exit nerve root complications in interbody fusion performed through the transforaminal transkambin route. It is a challenge to safely insert a minimum 7–13 mm outer diameter working cannula in a disc space when the mid sagittal disc height is less than 5 mm without compression of the exiting nerve root. In our cohort, pre-operative mid sagittal CT middle disc height of less than 5 mm were included in the study, and hence most if not all of our patients would be relatively contraindicated for the transforaminal approach transkambin endoscopic interbody fusion procedure [24,28]. We performed an approach that bears a greater resemblance to the interlaminar approach with posterolateral transforaminal interbody fusion in our cohort of patients. We took down the facet joint with endoscopic diamond drill. Care was taken to drill the last cortical layer dorsal to the exiting nerve root, so as not to cause any exiting nerve root dysesthesia. With the extra room created after facet resection, we were able to advance the working cannula towards the disc space without significant compression of the exit nerve root when performing the steps of interbody fusion. Therefore, despite the fact that we included all patients with severe collapsed disc space, no exiting nerve root dysesthesia or subsidence occurred in our series. 

In conventional open or microscopic tubular transforaminal lumbar interbody fusion, difficulty is presented by good end plate preparation deep in the intervertebral disc space due to limitations in the visualization of end plates in the collapsed disc space with associated syndesmophytes and dense adhesion around the intervertebral disc. To open up the collapsed disc space, surgeons have used various pieces of equipment, including pedicle screws with distractors and lamina spreaders placed on remnant bony structures, in order to create space for the spinal instruments to prepare the collapsed disc space. Pedicle screw distraction can lead to the loosening of the pedicle screws, and lamina spreaders can cause unnecessary fracture and damage to bony structures while performing end plate preparations. Full endoscopic Endo TLIF can help us to overcome these barriers. The beveled working cannula is important in severe collapsed disc cases in two ways: (1) it protects the neural elements out of harm’s way, creating a “tunnel” through which we can direct the endoscopic drill and sharp equipment directly into the disc space and perform endoscopic disc drilling safely. This facilitates the opening of a tight and collapsed entrance to the intervertebral disc space; (2) the tip of the working cannula can be used to straddle the intervertebral disc space in order to keep the collapsed disc space open and allow the endoscopic equipment to be passed smoothly into the intervertebral disc space for endplate preparation (Figure 8 and Figure 9) With the endoscopic vision at the distal tip of the endoscope, we can denude the disc and cartilage of the end plate from the posterior annulus to the anterior annulus just short of the anterior longitudinal ligament under direct magnified endoscopic visualization. As we are able to perform an optimal disc preparation without violating the end plate, we are able to better mobilize vertebra bodies adjacent to the disc space. Such increased mobility of the vertebra allows greater restoration of disc height even in cases of severe collapsed intervertebral disc space. 

In comparison to biportal endoscopic and open transforaminal interbody fusion techniques, which often require an assistant in the surgery for neural retraction, the working cannula acts as our neural retraction device and irrigation fluid, providing constant clarity of endoscopic vision during surgery. Therefore, no surgical assistant was required when performing the endoscopic fusion procedure. This would potentially reduce the overall cost of surgery and allow more efficient deployment of manpower resources. 

In severe disc collapsed lumbar spinal segments, we found that there was often dense adhesion between the degenerated disc and the neural elements, which could increase the chance of dura injury. With our technique, we gradually reduced adhesion through radiofrequency ablation around the neural elements. We used the working cannula to directly access the disc space while retracting the neural elements out of harm’s way. There was one case of incidental durotomy in our series. In that patient, there was no significant sequelae after the dura patch blocking repair [20]. This incidental durotomy rate is comparable to the incidental durotomy rate of other endoscopic techniques (0–8.6%), despite the significant neural adhesion and collapsed disc space in our series [20,29,30]. There were several other different complications reported in uniportal and biportal endoscopic procedures, with the common ones being transient paresthesia (1–3%), epidural hematoma, with a revision rate of 1.9%, and headache (1–2%) [31,32,33,34,35,36,37,38]. We had no other complications in our series, but we needed to be cautious in the interpretation of our results, as this technique is relatively new, and no long-term data is available for its results.

In Endo TLIF, we used a corridor between the well-described conventional transforaminal and interlaminar endoscopic approaches [9,39]. After we performed facetectomy, we found that the endoscopic view of spinal anatomy was more similar to interlaminar endoscopy than transforaminal anatomy (Figure 1). Nevertheless, there was a steep learning curve, as the operative view in this intermediate route can be disorientating for endoscopic spine surgeons. There were additional challenges in performing Endo-TLIF in the collapsed disc space, such as proximity of the exiting nerve root to the intervertebral disc space, the non-parallel end plates of the caudal and cephalad vertebral bodies, and the decreased size of the foramen in the concave part of the deformity. Endoscopic surgeons needed to pay special attention to these pathological anatomical variations in order to avoid surgical complications. Hence, in order to achieve consistent good results in Endo-TLIF, familiarity of lumbar endoscopic unilateral laminotomy with bilateral decompression would be necessary before attempting to perform Endo-TLIF. Navigation at various stages of the fusion procedure might help in orientation under spinal endoscopy in order to flatten the learning curve [40,41,42]. In our series, we performed intermittent checks using intraoperative fluoroscopy at various stages of procedure. 

There was significant improvement in the CT coronal and sagittal focal angle for Endo-TLIF cases, which decreased the effect of coronal wedging and sagittal kyphosis caused by severe disc collapse on the affected lumbar segment. However, we do not have evidence or advocate Endo-TLIF for severe scoliosis deformity of more than 20 degrees. In those cases, more soft tissue and bony releases, as well as more levels of fusion, might be necessary; hence, Endo-TLIF would not be the first choice of surgery in these cases [43].

We had clinically favorable and statistically significant improvements in pain score in our case series, we felt that this was a result of the usage of the same skin wound as the ipsilateral upper pedicle screw to approach the isthmus for facet resection, leading to less soft tissue damage and minimal neural retraction during the procedure, which could be the reason for the improved outcomes. However, as this technique is in the early stages of development, long-term clinical data were not available for more granular evaluation of this technique. 

Despite the discussion of the advantages of Endo-TLIF in comparison with transforaminal transkambin interbody fusion in severe collapsed disc space, transforaminal transkambin endoscopic interbody fusion has several advantages in terms of the preservation of soft tissue and facet joint and the surgery could be done under local anesthesia and moderate sedation. This is especially important in patients who have significant comorbidities. Our cohort of patients all required general anesthesia in their surgeries. Although we managed to harvest the facet joint as autograft in Endo-TLIF, the autograft obtained was insufficient compared to open or microscopic tubular procedures. Therefore, we needed to either harvest autograft from the posterior iliac crest, or we needed to use allograft and bone substitutes. In our cohort, we opted to use allograft bone to mix with autograft to promote fusion. Overall, there is no one technique that has been proven superior to others in all circumstances; therefore, surgeons need to be aware of the pros and cons of each technique before deciding on which technique to adopt to perform spinal fusion. 

## 6. Limitations

The data was obtained as a retrospective evaluation of patients who had undergone Endo-TLIF with severe collapsed disc space. There could be inherent selection and performance bias in the study. Pre-operative data such as comorbidities, Charlson Morrison Index, and length of operation time were not collected, which might have introduced confounders into the study. We limited these confounding factors by keeping the same team of anesthetists and surgeons throughout all of the operations performed in the data set. The follow up was relatively short and we continued to follow up on these patients with a view to evaluating the long-term results in the future. There was no comparative open surgery arm in this study, as the authors did not perform open spinal fusion for lumbar segments with severe collapsed disc during the study period. An independent radiologist assessment of the radiological parameters could minimize radiological assessment bias, and we limited bias by using fixed radiological landmarks for assessment. A prospective study and randomized controlled trial would be more suited to eliminating these biases. However, it would be difficult, given that we included patients with very specific criteria in order to evaluate the effect of Endo-TLIF in patients with severe foraminal stenosis secondary to severe collapsed disc space. 

## 7. Conclusions

Uniportal Endoscopic Posterolateral Lumbar Interbody Fusion in well selected patients with severe foraminal stenosis secondary to severe collapsed disc space significantly relieved patients’ pain and restored disc height without early subsidence or exiting nerve root dysesthesia in our cohort of patients. 

## Figures and Tables

**Table 1:** Basic demographics Computer Tomographic lumbar spine and clinical results of the cohort of patients who underwent Endo-TLIF.

	Mean	Range/(SD)	*p* Value	Remarks
Age (years)	68	54 to 78		
Levels				30 levels in 27 patients
Sex				9 male, 18 female
Follow up (months)	12	6 to 19		
Pre-operative VAS	6.23	5 to 7		
Post-operative VAS at 1 week	3.7	2 to 5		
Post-operative VAS at 3 months	3.03	1 to 4		
Post-operative VAS at final follow up	1.87	1 to 3		
Pre-op VAS-post 1 week VAS	2.53	1.137 (SD)	0.000	
Pre-op VAS-post 3 month VAS	3.20	0.890 (SD)	0.000	
Pre-op VAS-final VAS	4.37	1.033 (SD)	0.000	
Mid coronal CT disc height (post-op-pre-op)	7.13	1.90 (SD)	0.000	
Mid sagittal CT anterior disc height (post-op-pre-op)	6.99	2.30 (SD)	0.000	
Mid sagittal CT middle disc height (post-op-pre-op)	6.28	1.43 (SD)	0.000	
Mid sagittal CT posterior disc height (post-op-pre-op)	5.12	1.79 (SD)	0.000	
Mid coronal CT Coronal Wedge Angle (post-op-pre-op)	2.35	4.73 (SD)	0.012	
Mid sagittal CT focal segmental angle (post-op-pre-op)	1.98	4.69 (SD)	0.028	
